# On the way to Hepatitis C elimination in the Republic of Georgia—Barriers and facilitators for people who inject drugs for engaging in the treatment program: A formative qualitative study

**DOI:** 10.1371/journal.pone.0216123

**Published:** 2019-04-29

**Authors:** Ivdity Chikovani, Danielle C. Ompad, Maia Uchaneishvili, Lela Sulaberidze, Ketevan Sikharulidze, Holly Hagan, Nancy L. Van Devanter

**Affiliations:** 1 Curatio International Foundation, Tbilisi, Georgia; 2 College of Global Public Health, New York University, New York, NY, United States of America; 3 Center for Drug Use and HIV Research, New York University, New York, NY, United States of America; 4 Rory Meyers College of Nursing, New York University, New York, NY, United States of America; University of Cincinnati College of Medicine, UNITED STATES

## Abstract

Hepatitis C virus (HCV) infection is a significant public health concern worldwide. Georgia is among the countries with a high burden of HCV infection. People who inject drugs (PWID) have the highest burden of infection in Georgia. In 2015, the Government of Georgia, with partners’ support, initiated one of the world’s first Hepatitis C Elimination Programs. Despite notable progress, challenges to achieving targets persist. This qualitative study is aimed to better understand some of the barriers and facilitators to HCV testing and treatment services for PWID to inform HCV treatment policies and practices. The study instrument examined social, structural, and individual factors influencing HCV testing and treatment practices. We started with key informant interviews to guide the study instrument development and compare the study findings against health care planners’ and health care providers’ views. Forty PWID with various HCV testing and treatment experiences were recruited through the snowball method. The study found that along with structural factors such as political commitment, co-financing of diagnostic and monitoring tests, and friendly clinic environments, knowledge about HCV infection and elimination program benefits, and support from family and peers also play facilitating roles in accessing testing and treatment services. On the other hand, inability to co-pay for diagnostic tests, fear of side effects associated with treatment, poor knowledge about HCV infection, and lack of social support hampered testing and treatment practices among PWID. Findings from this study are important for increasing the effectiveness of this unique program that targets a population at high risk of HCV infection.

## Introduction

Hepatitis C virus (HCV) infection is a significant public health concern worldwide. An estimated 13 million people are infected with HCV in the European region [1]. People who inject drugs (PWID) are the main drivers of the HCV epidemic. It has been estimated that 64.7% (56.6% -72%) of PWID are exposed to HCV in Eastern Europe, which has the highest prevalence across the regions [2]. Georgia is among the countries with a high burden of HCV infection. A national population based survey conducted in 2015 found that 7.7% of the general population is anti-HCV positive and 5.4% are HCV RNA positive [3]. The study also revealed that use of injection drugs accounted for more than one third of cases among the general population [4]. Similar to other countries, PWID in Georgia are particularly vulnerable to HCV infection due to risky behaviors and exposure to structural and environmental risk factors. Approximately 65%-75% of PWID in Georgia are HCV antibody positive [5].

HCV treatment was first introduced to Georgia in 2011 through a program supported by the Global Fund to Fight AIDS, Tuberculosis and Malaria (“The Global Fund”) [6]. Initially, a combination therapy with pegylated interferon and ribavirin was available to HIV/HCV co-infected individuals at no cost [7]. In 2013, the Government of Georgia introduced free HCV treatment for prisoners and offered reduced price HCV treatment to the general population at a 60% discount rate [6,7].

In April 2015, the Government of Georgia and partners (i.e., the U.S. Centers for Disease Control and Prevention, World Health Organization, Gilead Sciences, The Global Fund, Emory University [USA], and Bristol University [UK]) initiated one of the world’s first Hepatitis C Elimination Programs with the goal of 90% reduction in HCV prevalence by 2020 [3,8]. Gilead Sciences, the pharmaceutical company that produces direct acting antiviral (DAA) HCV treatments, agreed to provide initial 5,000 courses of the antiviral medication sofosbuvir (Sovaldi) free-of-charge to support the program [3,7]. Patients with severe liver disease (i.e., METAVIR score F3 or F4) were prioritized to receive treatment during the first year of the program. The initial treatment regimens consisted of sofosbuvir in combination with pegylated interferon and ribavirin [8]. By February 2016, Gilead Sciences again agreed to provide 20,000 treatment courses of ledipasvir-sofosbuvir (Harvoni) annually at no cost [3] and the patients began receiving the new DAA regimen. The national program still uses interferon/ribavirin containing regimens in certain circumstances with the goal of eliminating interferon containing regimens and using of all-oral DAAs. In the second phase of the program, the severe liver disease criterion was abolished and as of today the program is accessible to all citizens of Georgia with chronic HCV infection [8].

By way of process, after screening for HCV infection, individuals positive for anti-HCV antibodies undergo confirmatory testing to determine active HCV infection by quantitative HCV nucleic acid test (NAT) or HCV core antigen test. If the diagnosis is confirmed further tests are required to determine liver fibrosis status and HCV genotyping. Number of tests are conducted during the course of treatment to monitor treatment progress and at the end to determine the treatment outcome.

Pre-treatment diagnostics, treatment monitoring, and post-treatment laboratory tests were covered by the program and local governments with some co-financing required from patients. Since the beginning of the program implementation socially vulnerable patients and war veterans have been co-financed up to 70% by the program and up to 30% by local municipalities so they receive completely free testing services. As for the rest of the population, cost sharing for diagnostics, monitoring, and post-treatment tests across the years is presented in Table 1 below.

**Table 1:** HCV diagnostics, treatment monitoring and post-treatment tests cost sharing.

Tests	Total costs (GEL)	2015	2016	2017	From Sept 2018
**Screening**					
Anti-HCV antibody testing		Patient– 0% Program -100%	Patient– 0% Program -100%	Patient– 0% Program -100%	Patient– 0% Program -100%
**Confirmation**					
HCV NAT	110	Patient—70% Program– 30%	Patient– 10%-60% Municipality 60%-10% Program– 30%	*From 1 Dec 2017*: Patient—0% Program– 100%	Patient -0% Program– 100%
HCV core antigen test	60 –from Dec 2017				
**Tests for inclusion:** Liver fibrosis status	375	Patient—70% Program– 30%	Patient– 10%-60% Municipality 60%-10% Program– 30%	Patient– 70% Program– 30%	Patient– 70% (max 160 GEL) Program– 30%
Further examination including HCV Genotyping	140	Patient—70% Program– 30%	Patient– 10%-60% Municipality 60%-10% Program– 30%	Patient– 70% Program– 30%	Patient– 0% Program– 100%
**Treatment monitoring tests**	300–500	Patient—70% Program– 30%	Patient– 10%-60% Municipality 60%-10% Program– 30%	Patient– 70% Program– 30%	Patient– 70% Program– 30%
**Post treatment test and consultation**	130	Patient—70% Program– 30%	*From mid 2016*: Patient– 0% Program -100%	Patient– 0% Program -100%	Patient– 0% Program -100%
**All tests for socially vulnerable population and war veterans**		Program– 70% Municipality– 30%			

GEL = Georgian Lari

NAT = Nucleic Acid Test

Drugs for HCV treatment were, and continue to be, fully covered by the program. With the goal of achieving 90% reduction in HCV prevalence by 2020, Georgia’s Hepatitis C Elimination Strategic Plan outlines the following elimination goals: (1) testing 90% of HCV-infected people for their infection, 2) treating 95% of the patients with chronic HCV infection, and 3) curing 95% of the patients treated of their HCV infection [8].

Georgia made substantial progress in the first year of the Elimination Program. Between April 2015 and April 2016, 27,392 people with HCV were enrolled in the program and 8,448 initiated treatment [3]. This translates to a more than 400% increase over the number of patients treated in the previous four years [3]. Yet, as is the case with any new larger-scale program, implementation can be challenging, especially with PWID. Despite evidence that this population can be successfully treated for HCV, the literature also describes low HCV treatment uptake among PWID and challenges associated with engaging them in HCV treatment [9,10].

This qualitative study aims to better understand the barriers and facilitators to HCV testing and treatment services for PWID in order to inform HCV treatment policies and practices in Georgia. Specifically, the research objectives were to (1) identify the societal, structural, and individual barriers and facilitators to HCV screening, completing diagnostic testing, and initiating HCV treatment services among PWID and (2) examine the perceived risk of HCV re-infection and its consequences among PWID.

## Methods

### Conceptual framework

A conceptual framework based on the health service utilization framework developed by Anderson & Newman (2005) was used to inform the PWID in-depth interview guide development. The health service utilization framework posits that health service utilization is influenced by characteristics of the health services, societal norms, and individual factors [11].

**Fig 1:**
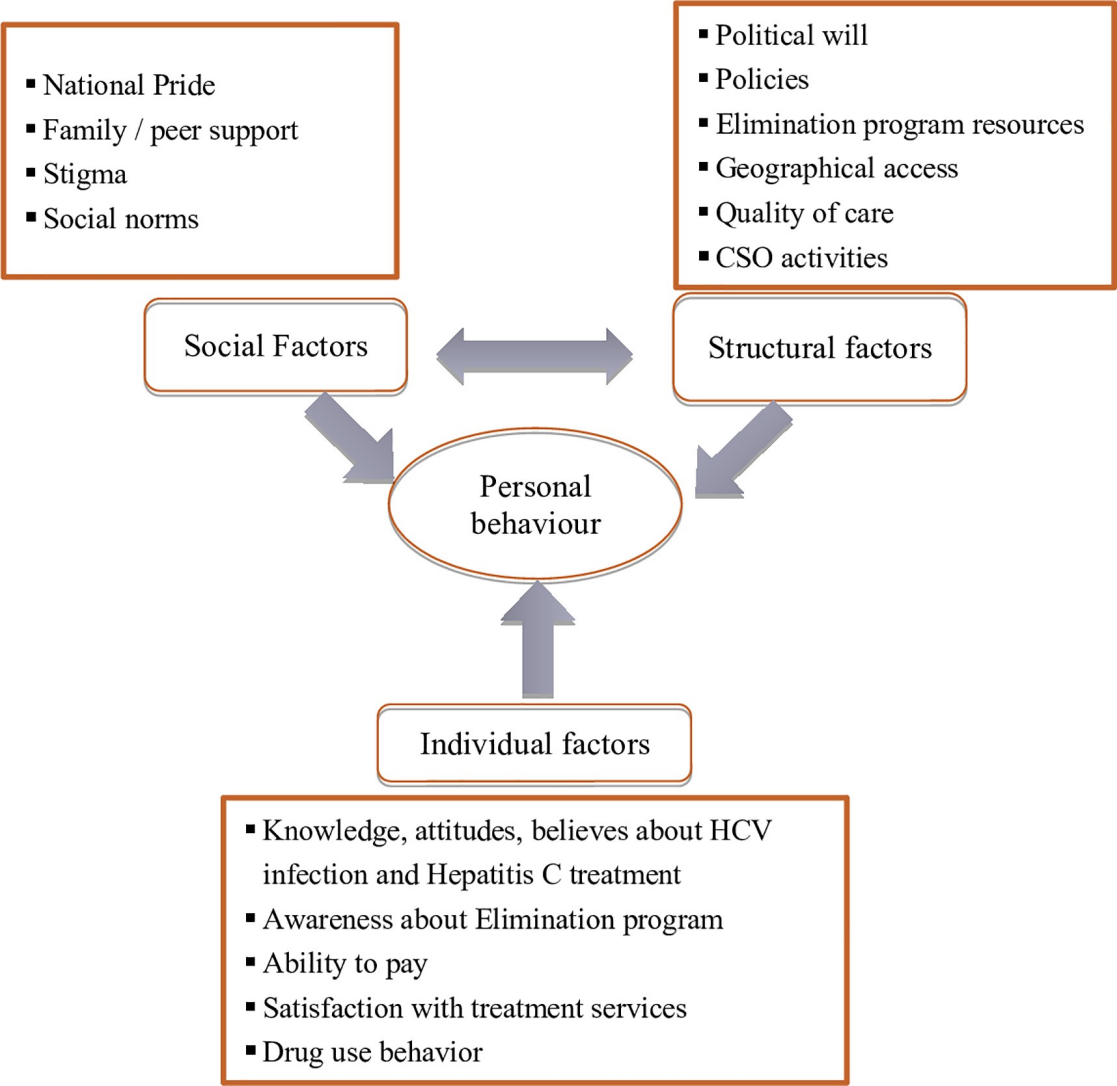
Health service utilization conceptual framework (modified Anderson and Newman, 2005).

We modified the framework and identified social, structural, and individual factors that may act as barriers or facilitators to using testing and treatment services (Fig 1). Social factors include family and community support, stigma, and peer influence as well as other social norms or attitudes that may influence a PWID’s decision to seek treatment, such as national pride in the Hepatitis C Elimination Program. Structural factors were defined as those over which a person has little control such as political will, policies, program resources, financial and geographical access barriers to service use, quality of care, and civil society organizations CSO activities. Individual factors include knowledge, attitudes, and beliefs regarding HCV infection, the Elimination Program in general, and HCV treatment specifically. Individual factors also include patient motivation and willingness, general lifestyle and drug use behavior, ability to pay, and satisfaction with services. Social and structural factors interact with each other and together influence individual factors.

### Sample and recruitment

Prior to the recruitment of PWID we conducted key informant interviews with the individuals who had first-hand knowledge about the Hepatitis C Elimination Program implementation, successes, and challenges. The purpose of these interviews was to guide the PWID data collection tool development and complement the study findings by examining the views of health care planners and health care providers on policies, elimination program resources, and other structural factors. The key informants were identified through a snow-ball method. We interviewed representatives from the Ministry of Labour, Health and Social Affairs (MoLHSA), HCV testing and treatment services, and Civil Society Organizations (CSOs) involved in harm reduction activities, who had particularly informed perspectives on the research topic. In total, seven key informants were interviewed face-to face by lead researchers in April 2016. This number was sufficient to gain a broad perspective of a situation from the representatives of divergent groups involved in the Hepatitis C program development and implementation.

PWID were recruited from six cities of Georgia (i.e., Tbilisi, Kutaisi, Batumi, Zugdidi, Telavi, and Gori). The target sample size was 40 PWID. We note that sample sizes in the 10s of participants are par for the course with qualitative studies [12]. Indeed, 40 is robust for a qualitative study. The concept of “saturation”–i.e., interview to redundancy–is often mentioned when discussing sample sizes in qualitative studies [13]. After 40 PWIDs had been recruited and their interviews reviewed, coded, and analyzed, the team discuss whether there was a need for additional interviews. Given the breadth and depth of the information collected through those 40 interviews as well as redundancy in perspectives on the processes of interest, we concluded that our sample reached saturation with respect to our research questions.

Eligibility criteria required that participants be 18 years of age or older, able to communicate in Georgian, and have injected drugs at least once during the six months prior to the study. PWID were recruited through harm reduction services using snowball sampling. In each harm reduction center, initial seeds were recruited and were asked to bring their peers to the study. The participants were especially encouraged to invite female injecting drug users. The selection criteria also required participants to represent the following subgroups: 1) PWID that have completed HCV treatment; 2) PWID undergoing a course of treatment; 3) PWID who were aware of their HCV status but were not receiving treatment; 4) PWID who were not aware of their status (i.e., have not been tested for the past 5 years); and 5) PWID who initiated treatment but interrupted before completion.

The study protocol was approved by the Institutional Review Board of the Infectious Diseases, AIDS and Clinical Immunology Research Center of Georgia (OHPR # IRB00006106).

### Data collection

The field work took place in June and July, 2016 during the second phase of the Hepatitis C Elimination Program. Experienced interviewers carried out face-to-face in-depth interviews with PWID in a private setting. Participants provided written informed consent for participation in the study. Semi-structured interview guides included open-ended questions and follow-up questions with probes relevant to the PWID experience. Demographic information was collected at the beginning of the interview. Interviews were audio recorded if the respondents granted their permission. Only one respondent refused to be audio-recorded. In this case detailed notes were taken by another data collector. Remuneration of 25 Georgian Lari (11 USD) was given to PWID respondents for their participation in the study.

### Data analysis

Interviews were audio-taped with participants’ permission, transcribed verbatim, and translated into English for analysis. Three members of the research team conducted an initial reading of all the transcripts to identify patterns and initial themes emerging from the data and themes that relate to the conceptual framework. After the initial reading, the research team utilized constant comparison to further develop a coding structure and a detailed code book. Two researchers coded all transcripts using a qualitative software QSR-Nvivo 11.4. Additional codes that emerged were discussed as they came up and were added to the codebook upon agreement. The complete set of coded transcripts was reviewed by one researcher for discrepancies and inconsistencies. Any differences in the coding were resolved through group discussions, review of the transcripts, and re-coding. Thematic analysis was conducted according to the conceptual framework.

## Results

In total, 40 current PWID participated in the study. Eight respondents had already completed HCV treatment, ten respondents were currently being treated for HCV, eighteen respondents were not involved in the program of which seven were not aware of their HCV status, and four respondents were on the waiting list for HCV treatment. The study failed to recruit any respondent who initiated and interrupted treatment. Demographic characteristics of the sample are presented in Table 2.

**Table 2:** Demographic characteristics of the sample.

Characteristic	N
Gender	
Male	39
Female	1
Age	
45 years and older	23
26–44 years	14
25 years and younger	3
Injecting drugs	
10 years and more	35
less than 10 years	5

The results are structured as follows: we first present facilitators and barriers of the decision to seek treatment and then adherence to treatment. Facilitators and barriers are further categorized by social, structural and individual factors. Finally, we present HCV re-infection risks among PWID. Findings from the key informants are presented along with those from the PWID.

### Facilitators of the decision to seek HCV treatment

#### Political support and media campaign (structural)

The Hepatitis C Elimination Program received strong political support from its early stages. As mentioned by the key informants, the Program became one of the most frequently cited topics by high government officials in their media appearances. The Program has been considered as one of the successes of Georgia health care system in 2015–2016. Key informants and PWID participants all mentioned the crucial role of media in advertising the Hepatitis C Elimination program.

#### Hepatitis C elimination program as a national pride (social)

Availability of such an expensive program (in the range of 60,000 to 120,000 USD per patient) at almost no cost to the patients was acknowledged as one of the leading factors that influenced PWIDs’ decisions to seek and complete treatment. Respondents admitted that they are “fortunate” that such program is available in the country.

“This is a huge thing done by the state to me, an ordinary person having an infectious disease. This is the same as having a new chance to live because it is a rather expensive program. I am fully aware of how much it costs, what is the price of the flacon of the medicine. I could not afford it. I had already gotten used to the fact that I had an incurable disease before the program was launched.” (Male PWID from Zugdidi)

“To us, to the patients this was so unimaginable that we were ready to tolerate everything. We tolerate everything to bring the treatment course to an end.” (Male PWID from Tbilisi)

Key stakeholders, treatment service providers, harm reduction network representatives also prized the program and consider it to be “unique” and not only valuable for Georgia but globally.

“Hepatitis C elimination program is an excellent opportunity for the population of our country to receive treatment using new generation, effective, and very expensive drugs. Many years ago, even during our advocacy efforts, we could not imagine such universal access to expensive medications, which are vitally important and grant patients a higher chance of being cured.” (Key informant)

“This program is important from the global perspective, and will set a precedent by eradicating the disease” (Key informant)

All key informants cited that the current treatment regimen has fewer side effects and better treatment outcomes. They also mentioned that news of successfully treated cases rapidly disseminated among patients’ networks, increasing the credibility of the program.

#### Knowledge about the HCV infection (individual)

PWID described HCV disease as a “liver disease leading to a cirrhosis,” and “silent death.” They were well aware of how HCV was transmitted as well as risk behaviors associated with spreading the virus such as sharing of needles and syringes. PWID described other modes of transmission including dental procedures and sexual contact. Participants mostly associated the disease with symptoms such as “fatigue, weight loss, jaundice;” however, very few mentioned that the disease could be asymptomatic. The participants, including those who were not involved in the program, believed that the disease could be cured, only one PWID thought that the disease is incurable.

#### Referral to the program and Public Financial support (structural)

According to the key informants, CSOs played a significant role in facilitating PWID referral to the HCV elimination program services. Harm reduction networks were actively involved in directing patients to treatment sites. The information was also widely spread by peer educators:

“The network of peer educators works very well. The information is transmitted rather quickly by word of mouth, sometimes I say something to a patient and couple of days later some other person comes and repeats my words. They spread this information rather quickly.” (Key informant)

Respondents from the capital city frequently mentioned the contributions of CSOs in covering diagnostic test expenses. Tbilisi patients appeared to be in a better position due to significant contributions from the Mayor’s office that, at the time of the study since 2016 have been covering 60% of diagnostic and monitoring test costs in addition to the program co-financing of 30%. Respondents also admitted that even though they needed to pay some amount for clinical diagnostics and laboratory tests to monitor treatment outcomes, compared to the complete cost of HCV treatment, theirs was a small share.

“Yes, you will find dissatisfied people everywhere but first they should look at the program, the quality and the price of the medicines they get, the amount the state pays for them. Compared to that, we pay a small portion. It is a minor share we pay, so let nobody say that this is a big amount for the tests.” (Male PWID from Zugdidi)

#### Social support from family and friends (social)

The support of family members, relatives and friends was considered to be a very important factor influencing patients’ decision to seek treatment. All patients, regardless of their desire and current involvement in HCV Elimination Program, admitted that such support is crucial.

“I would not have joined the program had my mother and family not insisted on it.” (Male PWID from Kutaisi)

“It is rather important. For example, my mother visited the Mayor’s Office as well as other places for the documents. I would not have been able to do that alone” (Male PWID from Kutaisi)

One participant who is no longer involved in treatment reported that if his family members had more information on the program and insisted on his treatment, he would probably have sought it. Another participant not currently involved in the program said that he “needs someone to take him to treatment,” and his family members have a difficulty providing such support.

### Barriers for decision to seek HCV treatment

#### Exemption from the program (structural)

Of the eighteen respondents who were not enrolled in the program, eleven reported that they did not qualify for the program based on the initial enrolment criteria. The patients regrettably admitted, “This is sad, because the treatment has been provided for free.” During the period when the interviews were conducted, severe liver damage (i.e., Metavir score F3 or F4) as a precondition for treatment had already been abolished. However, some of the patients did not know about these changes while some of those who knew were still planning to apply for treatment.

#### Ability to pay (individual)

As mentioned above, expenses for HCV screening were fully covered by the program, while confirmatory tests and tests needed for inclusion into the program were partially covered by the program (up to 30%) with co-sharing from the patients and local governments for the patients living in their municipalities. Additional tests are needed during treatment monitoring which also require patient contributions. As the respondents did not make clear distinctions between financial barriers associated with diagnostic, pre-treatment, and monitoring tests, we present these findings all together in the ability to pay section under the barriers to HCV treatment adherence.

The share of co-financing from local governments varied by municipality, resulting in some degree of inequality between residents of different geographic areas. During interviewing period in 2016, patients in the regions had to pay higher amounts than Tbilisi residents due to smaller contributions in the provinces from local authorities compared to the Tbilisi Mayor’s office. The patient’s co-payment ranged from 350 to 600 GEL (145–250 USD) for pre-treatment and monitoring laboratory diagnostics. To overcome this barrier, some PWID were trying to find ways to be registered as Tbilisi residents.

“We know about cases when patients registered in Tbilisi to receive more affordable treatment.” (Key informant)

Several cases were identified where patients who did not seek HCV testing stated that they could not afford the costs associated with treatment:

“I also think that I may need money for treatment but I have rather serious problems. It is not possible for me to start the treatment course now.”(Male PWID from Rustavi, not enrolled in the program)

#### Geographical access (structural)

Geographic barriers were mentioned as an obstacle for the patients living in the Kakheti region, where at the time of data collection, treatment facilities did not exist. This lack of facilities created an additional financial burden to PWID in that region because of associated travel costs to Tbilisi.

#### Knowledge about HCV, risk perception, and difficulty initiating treatment (individual)

Among the entire sample, only six respondents (15%) have never been tested for HCV in their lifetime. Inadequate knowledge about the disease and lack of awareness that HCV infection could be asymptomatic discouraged PWID from seeking HCV testing. Others thought that HCV testing is unnecessary because they considered themselves to be at low risk of contracting HCV due to safe injecting practices. At the same time, some admitted that if they were infected then they would seek treatment.

“I think: Why should I go if nothing bothers me? I do not have to hurry. I will go there tomorrow; I will go there the day after tomorrow. Then you forget about it”. (Male PWID from Tbilisi, not enrolled in the program)

“If I had C hepatitis I would feel something, would not I? I think so … I cannot be 100% sure but I still think that I do not have it” (Male PWID from Tbilisi, not enrolled in the program)

Some patients expressed low interest in their health and the possibility of treatment. They were more preoccupied with other problems, even though they did not rule out the disease. Some expressed nihilism about the disease and its consequence:

“Maybe I am afraid but at that moment I do not think about that, I think about injecting drugs. …. I say to myself ‘let it kill me whenever it decides to do so’” (Male PWID from Rustavi, not enrolled in the program)

Some patients found it difficult to initiate treatment: “I just need to begin it,” and “I need someone to push me.”

Interestingly, these PWID were in contact with their peers who were under treatment and were aware of the program. Some of them had heard about unsuccessful cases, and were discouraged from initiating treatment.

#### Fear of test results and treatment (individual)

Few PWID reported fear of learning that they are HCV positive as a barrier from getting tested:

“I may be afraid of that most. If I go there and they tell me that I have a terrible condition, when I know that I have no health problems, this will cause depression of course.” (Male PWID from Tbilisi, not enrolled in the program)

Some participants not yet enrolled in HCV treatment believed that treatment may harm more than cure. Fear of side effects and damage to their liver discouraged them from initiating treatment. Treatment with Interferon was not attractive to some of those who were not looking for treatment; however, some were exploring the possibility of treatment abroad where “interferon-free” treatment is available.

“I am afraid to start treatment. I saw some people feeling bad because of Interferon. I used to think I could die because of the treatment. This is fear, fear of death probably.” (Male PWID from Batumi, not enrolled in the program)

“I am afraid of the medicines they use here. I saw people who were on Interferon. They could hardly stand on their feet, they had fever.” (Male PWID from Tbilisi, not enrolled in the program)

Service providers also indicated that there are misconceptions about the side effects of interferon treatment that hamper treatment initiation. Providers describe several cases where patients were reluctant to initiate interferon treatment out of fear of its side effects. These misconceptions are rooted by negative experiences with interferon side effects that are shared among peers. Successful treatment outcomes with the interferon regimen play an important role in reducing these misconceptions.

“There are many rumors here about interferon and other drugs. They say it causes falling of hair and teeth. I explain to everyone that this is not the case, and I am an example of this.” (male PWID from Kutaisi)

PWID not currently involved in the program are curious about whether a new generation drugs has been introduced and what will happen to their health if they resume using drugs after HCV treatment; they are eager to learn about treatment outcomes from their peers.

#### Stigma (social)

All the participants, with the exception of one, did not feel stigmatized as a result of their positive HCV status. Among the participants currently not under treatment, none reported social stigma as a factor preventing them from treatment. Nevertheless, there are cases where participants do not wish to disclose their status and chose to hide it, because HCV is associated with drug injection.

“I do not want many people to know that I have Hepatitis C, this is what makes me uncomfortable.” (Male PWID from Kutaisi)

“I know many people who do not tell their families and receive treatment in secret…” (Male PWID from Tbilisi)

The study managed to enroll only one female PWID and she addressed her experience of being a female with HCV infection. Although she did not report any stigmatized attitudes from treatment service providers during the treatment process, making a decision to start treatment was difficult nonetheless.

“There is a tendency to stigmatize woman with Hepatitis C which causes them to feel discomfort to get treatment. Unless the organization (CSO) had offered I would not have been able to do that myself.” (Female PWID from Rustavi)

#### Skepticism about effectiveness of the program (individual)

In two cases the participants expressed a lack of confidence in the program:

“There are the following speculations: “Why would they (the government) do that to you?”, “they are helping us die,” “maybe this is some experiment?” (Male Batumi PWID, not enrolled in the program)

“But is the medicine reliable? Does it treat patients? Which other side effects does it have? … I do not even know the name of the medicine.” (Male Batumi PWID, not enrolled in the program)

According to the key informants, at the initial stages of the program implementation enrolment of individuals with severe liver damage did not deliver the desired results. Some patients with severe liver disease were not cured or died soon after treatment; news of such cases spread quickly through PWID networks. Moreover, patients and even health workers did not immediately understand why such an expensive program was offered to the Georgian population free of charge and were skeptical about it.

Stakeholders believed that the Government needs to spend more time explaining the advantages of the HCV elimination program to the country as well as what motives the pharmaceutical company might have. Clarifying that donating the drug is in the pharmaceutical company’s business interest would resolve the skepticism.

#### Programmatic challenges (structural)

Key informants identified several challenges that were encountered at the beginning of the program. The program start-up was preceded by an intensive preparatory stage, but doctors were informed that the program was to begin only one week prior to initiation of activities. Training was provided only after the program launched; however, since then doctors have received continuous technical support.

Key informants mentioned that, at some point, the program’s public relations campaign was so aggressive and mismatched with the program’s phases that it created problems with the patient flow and waiting lists. Due to very intensive advertisement in media, patients rushed to get treatment and it was difficult to manage the processes.

PWID respondents also indicated that such problems were gradually resolved in the process of the program implementation. The MoLHSA was responsive and eager to fix the problems in a timely manner to allow a smooth implementation of the program.

### Facilitators of HCV treatment adherence

#### Clinical environment (structural)

*Attitude of staff*. Eight out of the ten PWID respondents who completed treatment or were in the process of being treated described treatment sites to be safe environments with friendly and responsive staff. They mentioned that health professionals acknowledge the patients’ needs and are flexible in scheduling appointment times to ensure that the patient is seen. Participants pointed out that positive patient-provider relationships promote adherence to treatment. They described how health professionals are an important support in this process.

“The doctor greets you and talks to you in such a manner that you are pleased to visit the clinic.” (Male PWID from Tbilisi)

“The doctor also encouraged me and gave me hope. This was a big incentive to me.” (Male PWID from Kutaisi)

*Waiting times.* The patients recalled waiting times at the beginning of the program, with fewer lines now. They would describe 10–15 minutes waiting time in queues. The MoLHSA took steps to reduce the influx of patients to the facilities and started to manage the lines at the central level. They introduced a new mechanism by which a front office would schedule patients based on the clinics’ availability. Very few participants still mentioned lengthy waiting times during the treatment process; however, this was not considered as a barrier to receive treatment among patients.

*Pill taking in front of camera.* The program protocol requires taking pills in front of a camera in certain cases when there is suspicion that the treatment regime was violated.

In most of the cases, patients did not feel concerned about this. There was a threat of incarceration associated with injection drug use; however, participants were confident that the recordings would not be disclosed to the police or the public. Those who were involved in the methadone substitution program felt more relaxed about this feature, as methadone dosing in Georgia is also conducted in front of a camera. Similarly, the service providers confirmed that the patients did not object to taking pills in front of camera.

#### Quality of care (structural)

PWID talked about caring and responsive health professionals who were always ready to provide detailed answers to their questions. Doctors provided advice about taking care of themselves during the treatment process and warned about harmful behaviors. Respondents were confident that they were in the hands of qualified professionals and received appropriate care.

At the same time, patients (who had experience with interferon treatment) wished that they had a qualified provider to deal with mental health symptoms (e.g., irritability, depression, sleep disturbances) which were perceived to be quite frequent side effects of interferon treatment. HCV treatment service does not include consultations with mental health specialists qualified to provide such care.

#### Social support from family and friends (social)

For many patients, family members and friends provide invaluable emotional and practical support during the treatment process. Family members encouraged patients, reminding them to take their prescribed drugs and accompanying them to medical appointments.

“They provide incentives for living. When you have people who stand by your side you have hope.” (Male PWID from Batumi)

“Family support is important, very important. Not only in this regard. A family member, a spouse, may tell you something that will make you give up treatment, or the opposite, support you and make you think that it is worth to live.” (Male PWID from Batumi)

### Barriers to HCV treatment adherence

#### Ability to pay (individual)

Among the barriers to seek and remain in treatment, participants reported financial challenges in covering costs for tests. Apart from the HCV screening test, which is free under the program, a number of additional tests are required for confirmation testing, inclusion in the program, and treatment monitoring. At the time of data collection, the national program covered 30% of these costs and the rest were co-shared between the local authorities and patients. The patient’s co-payment ranged from 350 to 600 GEL (145–250 USD) for pre-treatment and treatment monitoring laboratory diagnostics. During the interviewing period in 2016, Tbilisi patients had to pay 10% of these costs due to higher contribution from the Tbilisi Mayor’s office compared to other municipalities. To overcome the financial barriers, some PWID were trying to find ways to be registered as Tbilisi residents.

“We know about cases when patients registered in Tbilisi to receive more affordable treatment.” (Key informant)

The co-payment was not affordable for some households. Several cases were identified where patients who did not seek HCV testing stated that they could not afford the costs associated with treatment.

The problem was more profound for those living far from the cities where treatment sites are located, due to additional transportation costs.

“I do not have anything to complain about myself but people are not able to pay for the tests. I basically mean people from provinces… I came across the cases when some patients could not afford tests and were not able to continue treatment.” (Male PWID from Rustavi)

For some patients, extra expenses before treatment appeared to be much bigger than anticipated. This was mainly for cases when the three months duration of HCV treatment was not sufficient to achieve cure.

At the beginning of the elimination program implementation, patients had to co-finance the final HCV NAT test needed to confirm the treatment outcome, which along with the consultation, costs 130 GEL (54 USD). As reported by key informants, due to financial difficulties, patients did not show up for this final test. This ultimately affected the treatment outcome data and the overall program performance statistics. After acknowledging this problem, the MoLHSA made a decision to fully reimburse the final HCV NAT test. This came into force at the beginning of the second phase of the elimination program, in mid-2016.

From January 2017, the Tbilisi Mayor’s office and other municipalities stopped co-financing HCV confirmatory and monitoring tests. This was partly due to a budget deficit and partly aimed to reduce inequality between Tbilisi and residents in other cities. One key informant viewed this as a positive, rather than a negative step towards creating motivation to adhere to treatment:

“When patients have some obligation to pay they feel more responsible during treatment. Moreover there was a significant difference between Tbilisi and other city residents which created a lot of complaints.” (Key informant)

#### Side effects (individual)

Patients who were treated with interferon experienced the side effects associated with this drug, mainly with the first injections. Common side effects include fever, fatigue, depression, anxiety, panic attacks, and insomnia. Patients were informed in advance by service providers about possible side effects and how to reduce them. However, in some cases, the side effects were more severe than expected, and patients indicated that only receiving information about side effects was not sufficient. Patients expressed fear that they could not tolerate taking this drug again if needed. Some patients refused to initiate treatment with interferon, opting to wait for new drugs before resuming treatment.

The MoLHSA representative mentioned that in the few months prior to the interview they encountered cases where patients interrupted treatment and resumed it later. To reduce the likelihood of such cases, the program added a policy to restrict re-enrolment into the program for one year for patients who stopped their treatment before completion of the regimen.

### Prevention of re-infection

Patients were well aware of HCV re-infection risks. They were advised by treatment service providers not to use drugs or to share injecting equipment. Some participants were firm in their decision not to engage in risk behaviors, to adopt a healthy lifestyle, and even to abandon drug injection following completion of treatment. Some drug users tried to shift to non-injection drugs, however about 60% thought that abandoning the use of injection drug was far from reality, “…if someone offers (drugs), this is a great temptation,” “…if I say ‘definitely no,’ that would be a lie.”

As they continue to inject drugs, respondents admitted to being at risk for reinfection with HCV. PWID articulated that such “failure” would be their fault. They were well aware of the risks associated with non-sterile injecting equipment use; however, in certain circumstances they may still use unsafe injection practices.

“We always have syringes from here (harm reduction program). But generally, of course there is a risk. ….If it happens again it will be because of our carelessness. More or less all of us know that we should not do that but…” (Male PWID from Zugdidi)

“It could happen maybe, when a person tells you that “yes, the syringe is new.” Moreover, not to offend them we do not ask whether the syringe is new and raise doubts … If I have the slightest doubt I will refuse–but who knows, he could be mistaken.” (male PWID from Tbilisi)

Some blamed dental clinics for transmitting HCV, which is “difficult to control” and poses risk for re-infection.

## Discussion

Georgia is poised to provide treatment to more than 120,000 individuals with chronic HCV infection, with the ultimate goal of reducing prevalence of HCV infection by 90% [8]. This is an unprecedented approach to implementing HCV treatment on such a large scale. From April 2015 through March 2018, Georgia’s HCV Elimination Program has managed to enroll 45,000 chronic HCV patients in treatment, of whom 29,000 achieved cure (i.e., sustained virologic response) [14].

Despite notable progress, challenges to achieving targets remain. A major challenge is that Georgia has a high a prevalence of injection drug use; according to a 2017 study about 52,000 adults or 1.41% of the general population injects drugs [15]. Moreover, PWID have the highest burden of infection in Georgia; more than 60% were HCV antibody positive as per a 2017 bio-behavioral study across seven major cities of Georgia [5]. A 2012 study among 216 active PWID in Tbilisi reported 92% were anti-HCV antibody positive and 82% were positive by HCV NAT [16]. Undiagnosed and untreated PWID may transmit HCV to other PWID as well as their sexual partners. Georgia’s National Strategy for Hepatitis C Elimination recognizes PWID as a key target group. To reach the Elimination Program goal, the national strategy outlines activities such as supporting access HCV treatment for PWID as well as promoting harm reduction to reduce disease incidence [8].

Our study provides a better understanding of PWID in the context of seeking and adhering to HCV treatment, which is critical to improving treatment uptake and retention. Factors influencing treatment seeking and adherence include structural, social, and individual factors. In terms of structural factors, political commitment, co-financing of diagnostic and monitoring tests, and friendly clinic environments were key facilitators for the Hepatitis C Elimination Program in Georgia. The study identified some programmatic gaps; however, they were profound largely at the beginning of the program and mostly created operational challenges for service providers, rather than influenced treatment seeking and adherence among patients.

The program received substantial political support starting from its launch and remains among the top health sector priorities in the country [8]. The study findings support the suggestion that strong political commitment plays a key role in smooth implementation of the program and its success so far. Other structural factors positively influencing treatment uptake were the roles of TV and harm reduction networks in advertising the program and referring patients to the treatment sites. The success of the campaign at initial stages even created problems due to rapid influx of new patients seeking treatment; however, the program quickly adapted to manage the situation.

Many participants described the relatively low cost for medical testing to monitor treatment response. Availability of co-financing from the program’s side for diagnostic and monitoring tests was critical to facilitating access to treatment services. However, the share to be paid by patients created a burden for some individuals, particularly in the provinces. At the time of data collection, local municipalities additionally co-financed monitoring tests for those seeking such financial support. Some of the key informants raised valid concerns that offering no-cost treatment might undermine the patients’ commitment to complete treatment. In addition, different co-financing offered by various municipalities created inequities in patients’ financial contributions. As a result, municipality co-financing was suspended starting in January 2017. Further policy changes in 2017 and 2018 included covering costs for the confirmatory test along with the final testing and HCV genotyping from the program budget. Current patients who are not under the poverty line need to pay about 125–155 USD (320–400 GEL) for pre-treatment, treatment monitoring and post-treatment tests. Whether this structural change has any influence on treatment seeking behavior or on treatment adherence is difficult to judge without further research. However, considering that average monthly income among PWID is within 40–120 USD (100–300 GEL) in Georgia the test costs may act as a barrier in access to treatment [5].

Additional financial barriers existed for those who were living in the regions where treatment services were not available. During 2017 and 2018, the number sites providing HCV treatment services more than doubled thereby reducing some existing geographical and financial barriers to treatment entry. Individuals who fall under the poverty line and war veterans were totally exempted from co-payments from the beginning of the program. Few of our respondents who qualified for the financial exemptions confirmed that treatment was completely free for them.

Our research identified social factors that affected access and adherence to treatment. Under this domain we included family and peer support, stigma, social norms, and other cultural factors. Social support was found to be essential in encouraging PWID to seek treatment and engaging them in treatment until completion, which is in line with the literature that examined the role of social context in treatment uptake and adherence [17]. Family and peer support could help PWID overcome structural barriers and positively influence personal behavior. Peer-to-peer support has been shown to increase treatment adherence [18]. Local experiences have also highlighted the important role of peers in maximizing treatment adherence: more than 200 PWID enrolled in HCV treatment during the first phase of the elimination program were followed by specially trained peer workers that resulted in 98% completion of the treatment course [19].

Stigma associated with injection drug use and HCV as a barrier to seek treatment has been documented in the literature [20]. Participants in our study did not mention experiencing stigmatized attitudes from health professionals at HCV treatment sites; however, more generalized stigma due to the association of HCV with injection drug use has been reported. This, along with lower prevalence of injecting drug use among females could be reasons for poor recruitment of female PWID in our study, as well as in other studies related to PWID in Georgia [5,15,19].

The majority of PWID respondents, as well as key informants and service providers, highly valued the program and admit that the country has received an extraordinary opportunity to benefit from it. The program represents a point of national pride and respondents expressed concern that failure of the program will show the country in a negative light. We speculate that such representation of the program could stimulate the service providers’ performance that, in turn, will positively affect patients’ behavior.

In general, a wide range of individual, patient-related factors influence the decision to seek treatment and [17,20]. study demonstrated that PWID in Georgia had a high degree of awareness of HCV treatment possibilities. At the same time, underscoring of the consequences of the disease, lack of knowledge that HCV can be asymptomatic, false perceptions that they are at low risk to contract the disease once they practice safe injection (i.e., use of sterile needle and syringe), and fear of being tested represented barriers at the stage of decision to get tested for HCV and enter treatment. The recent PWID bio-behavioral study in Georgia found that HCV testing remains inadequate–a serious impediment for the elimination program. The study showed that 26.5% of current PWID have never been tested for HCV. Among those who have never been tested, one third thought that they “do not need it,” another one third “do not think about it,” and 12% were afraid of the test results [5].

HCV drugs side effects may hamper the decision to start and stay in treatment; fear of side effects were mentioned by a few respondents as barriers to seeking treatment and this finding is also corroborated by the bio-behavioral study indicating that about 5.6% of those who were not on treatment refrained from it because of possible side-effects [5]. Inadequate management of mental health symptoms associated with HCV treatment was mentioned in our study and may be an obstacle in treatment continuation [21]. However, it is expected that the DAA, already in place in Georgia, will reduce this issue as fewer and less side effects are associated with these medications [22]. The study did not look at other mental health issues.

Once the patients are enrolled in treatment, they comply with the treatment regimen for the most part. This is largely supported by a friendly environment in the clinics as well as a responsive and caring staff that is critical to maintain the patients in treatment. The study is in line with the other research indicating that a friendly environment in the clinics, flexible service hours, and professionalism of the staff are important facilitators to treatment adherence [18].

Reinfection after cure could be another threat to the Hepatitis C Elimination Program. Enrollment into the Program does not require active PWID to quit injecting drugs. A majority of the study participants admitted that they will continue injecting drugs; therefore, the risk to of reinfection is real. The literature suggests that risk of reinfection among PWID was considerably lower than estimates of the risk of primary HCV infection among the same group [23]. Advice on reducing the risk of re-infection will be critical to minimize reinfection rates. This could be effectively delivered by harm reduction programs and peer educators [19]; however, another drawback is the poor uptake of harm reduction services by PWID in Georgia. According to the latest research reports only 26.8% of PWID have benefited from the needle and syringe exchange program [5].

## Study limitations

Our study is subject to several limitations. Although the study attempted to recruit female PWID, we were only able to enroll one female participant. In general recruitment of female PWID is challenging in Georgia due to lower prevalence of drug injection among females compared to males and high levels of stigma towards women who inject drugs leading them to be one of the most hidden subgroups [15,19]. The study also failed to enroll the PWID with a history of treatment interruption therefore our sample excludes views of this subset.

The respondents in the study were a convenience sample recruited through harm reduction service centers, therefore the findings should not be generalized to the PWID community in the country and other geographic areas. The views of the participants who agreed to participate in the qualitative study might be different from those who were unwilling to participate. Finally, PWID interviews were conducted during a limited time period, while some policy changes took place afterwards and therefore their effects were not captured by the study.

Despite these limitations, findings from this study are important for increasing the effectiveness of this unique program that is reaching a critical population at risk infected by HCV.

## Conclusion

This study provides important insights into the implementation of the Hepatitis C Elimination Program in Georgia and also highlights barriers and facilitators to HCV treatment initiation and completion. The Georgian program should enhance its outreach to PWID communities to encourage HCV testing and use of harm reduction services as well as to provide education about HCV and HCV treatment. This can be accomplished by continuing to leveraging PWID relationships with CSOs.

Co-financing for clinical diagnostics and laboratory tests is an essential element of the program, particularly for impoverished PWID. Ensuring that this program element is sustained at adequate levels across the country will be an important facilitator of treatment initiation and completion.

Despite some challenges the Georgian program is an example for other countries wishing to initiate HCV elimination programs.
